# Séroprévalence du virus de l'herpès humain-8 chez des patients VIH positif à l'hôpital général de Yaoundé – Cameroun

**DOI:** 10.11604/pamj.2015.20.69.5671

**Published:** 2015-01-26

**Authors:** Njiki Bikoï Jacky, Ndom Paul, Mupang Lilian, Agokeng Demanou Sylvie

**Affiliations:** 1 Faculté de Médecine et des Sciences Biomédicales, Université de Yaoundé 1, Yaoundé, Cameroun; 2 ONG SOCHIMIO Yaoundé, Cameroun; 3 Hôpital Provincial de Bertoua, Bertoua, Cameroun

**Keywords:** Séroprévalence, HHV8, IgG anti-HHV8, VIH, Sarcome de Kaposi, Seroprevalence, HHV8, anti-HHV8, HIV, Kaposi Sarcoma

## Abstract

L'épidémiologie de l'infection par le virus herpès humain de type 8 (HHV8) associée à celle à VIH, reste encore méconnue au Cameroun, bien que le pays soit considéré comme une zone endémique pour ces deux virus. L'objectif de ce travail était de ressortir le profil de la séroprévalence du HHV8 au sein de notre population d'étude. 57 personnes ont été recrutées à l'Hôpital Général de Yaoundé et suivies sur une durée 12 mois. Des anticorps IgG anti-HHV8 ont été déterminés par ELISA. Des paramètres autres, tels que l'âge, le sexe, le stade des maladies (SK et VIH/SIDA), le protocole ARV, ainsi que les taux de CD4 ont été utilisés pour déterminer les variables associées à la séropositivité au HHV8. Cette association a été évaluée par le test khi carré. La séroprévalence du HHV8 était de 90% dans notre population en début d'étude et de 74% douze mois plus tard, une séroprévalence qui restait élevée quelque soit le profil clinique, la tranche d'âge, le sexe ou le taux de CD4+ de l'individu. Aucune variable de l'étude n'était significativement associée à la séropositivité du HHV8. Le virus HHV8 semblait circuler au sein de notre population d'étude. Cependant l'on constate, douze mois plus tard, l'absence de manifestations cliniques du SK chez les patients VIH+ positifs, malgré des titres très élevés en IgG anti-HHV8.

## Introduction

Le sarcome de Kaposi (SK) a été décrit pour la première fois, en 1872, par une dermatologue hongrois, Moritz Kaposi [1]. Le sarcome de kaposi existe sous quatre formes : la forme classique est celle décrite initialement par Kaposi lui-même, touchant des hommes âgés de 50 à 70 ans, originaires de l'Europe de l'est et du pourtour méditerranéen [1]; la forme endémique est retrouvée, au début des années 60, dans les populations d'Afrique subsaharienne, atteignant des sujets plus jeunes [2]; la forme iatrogénique ou liée aux transplantations est associée aux patients recevant un traitement immunosuppresseur, suite à une transplantation d'organes [3]; la forme épidémique liée à l'infection à VIH [4]. C'est le cancer le plus fréquemment associé à l'infection au Virus de l'Immunodéficience Humaine (VIH) en Afrique [5]. Au Cameroun, en 2012, le SK représente 6,9% des cancers et est la néoplasie la plus associée au VIH/SIDA [6] et les traitements utilisés contre le SK sont la radiothérapie et la chimiothérapie, bien qu'étant lourds physiquement pour les malades VIH positif, car entraînent parfois une accentuation du déficit immunitaire et de la morbidité [7–9]. Le HHV-8 est un gammaherpès virus du genre Rhadinovirus. Découvert dans la lésion du KS en 1994 [10], ce virus est responsable de toutes les formes du sarcome de Kaposi au Cameroun [2, 11]. Cependant, peu de données existent sur le HHV-8 au Cameroun et plus est sur son association à l'infection à VIH. L'objectif de notre étude était de déterminer la séroprévalence du HHV8 dans notre cohorte et de discuter, à la lumière de la littérature, les données épidémiologiques, biologiques et cliniques associées à cette séroprévalence sur une période de suivi de 12 mois.

## Méthodes

Du 1er Juillet 2010 au 31 Décembre 2011, au sein l'Hôpital Général de Yaoundé (HGY), cette étude a été réalisée avec un effectif total de 57 personnes VIH positif, pris parmi les patients venus en consultation. 43 d'entre elles présentaient en plus une manifestation clinique du SK. C'était une étude cas-témoins. Les cas étaient recrutés parmi les personnes arrivant en consultation au sein du Service d'Oncologie Médicale, présentant des signes cliniques d'un sarcome de Kaposi confirmé associé au VIH et appartenant aux deux sexes. Les témoins ont été recrutés par la suite, appariés aux cas par l'âge, au moins un témoin par tranche d'âge. Ces témoins étaient pris parmi les patients du CAT de l'Hôpital Général de Yaoundé. La lecture au préalable de fiche de consentement éclairé a été faite aux participants, pour expliquer les objectifs de la recherche et les risques prévisibles encourus. La signature du participant fut apposée en bas de page afin de certifier avoir librement donné son consentement à participer à l'étude. Pour chaque participant à l'étude, nous avons relevé les données concernant l'âge, le sexe, en plus du stade du SK et du nombre de cure de chimiothérapie, pour ceux présentant un SK. Au sein du Service d'Oncologie Médicale où ces malades étaient suivis pour un SK, le nombre de cure de chimiothérapie Adriblastine-Bléomycine-Vincristine (ABV) recommandé était de 6 cycles, un toutes les trois semaines. Les patients retenus pour l'étude étaient constitués de 34 hommes pour 23 femmes, dont l'âge moyen était de 41,28±11,32 ans (de 22 à 69 ans), la tranche de 31 à 40 ans étant la plus représentée (39%).

On observait une nette prédominance du stade 4 (35 cas/61%) pour l'infection à VIH, et les combinaisons ARV les plus utilisées étaient 3TC - AZT -EFV et 3TC - AZT - NVP, respectivement 25% et 24%. Pour ceux avec un SK clinique, le stade de la maladie SK le plus rencontré était T1 (39%). La région du corps la plus touchée était celle sur les membres inférieurs (40 cas/70%) avec une très importante présence de macules cutanées. Chaque participant avait été suivi sur une durée 12 mois, et les données biologiques récoltées d'abord au moment de l'inclusion à l'étude (Temps 0 : T0) et puis après 12 mois (Temps 12 : T12). Les paramètres de l'étude ont été mesurés au moment de l'insertion à l'étude et 12 mois plus tard. Pour les patients avec un SK, ces paramètres furent mesurés avant le début du traitement ABV et six mois après arrêt de ce traitement. Pour chaque participant à l'étude, un examen physique a été fait, suivi d'une prise de sang dans un tube sec de 5ml, pour le dosage de taux de CD4^+^et de la titration des IgG anti-HHV8, ceci au moment de l'inclusion dans l'étude, et 12 mois plus tard. Le sang total a été analysé dans l'après-midi suivant le prélèvement pour déterminer le taux lymphocytaire de CD4^+^. Seuls les aliquots de sérums ont été conservés à-30°C jusqu'au moment du test sérologique pour la recherche des anticorps de type IgG anti-HHV8. Cette recherche a été effectuée par la technique ELISA indirecte (Advanced Biotechnologies Inc, KSV/HHV-8 IgG ELISA kit), selon les recommandations du fabricant. C'estait un test sérologique permettant la détection des anticorps spécifiques à l'antigène lytique du virus dans le sérum des patients. La stadification clinique du SK a été faite selon le modèle mis sur pied par l'Université de Californie (Los Angeles, USA) [12]. La stadification pour l'infection à VIH a, quant à elle, été faite suivant la définition du stade clinique du VIH chez l'adulte par l'OMS [13]. Le taux de CD4^+^mesuré, permettait de définir le degré d'immunodépression de notre série. Les analyses statistiques ont été réalisées avec le logiciel R version i386 3.1.0 et la valeur de p (p value) inférieure à 0,05 considérée comme significative, lors de l'association de la prévalence du SK dans notre population (obtenue ici grâce au titre en anticorps anti-HHV8) avec les autres paramètres de l'étude. L'étude avait au préalable obtenu la clairance éthique du Comité National d'Ethique du Cameroun en 2009.

## Résultats

Dans la population d'étude à T0, la prévalence globale de l'infection à HHV8 était de 90%, avec 64% de personnes ayant ce titre en anticorps bien supérieur à 3UI. A T12, cette séroprévalence régressait 74%, où seulement 26% de la population restaient à haut risque de transmission (>3UI).

***Séroprévalence du HHV8 chez les patients ayant uniquement une infection à VIH:*** au moment de l'insertion à l'étude (T0), la séroprévalence en IgG anti-HHV8 était de 79%, avec 43% de malades à risque de transmission. Douze mois plus tard, cette séroprévalence tombait à 57%, avec seulement 14% de malades à risque de transmission.

***Séroprévalence du HHV8 chez les patients présentant en plus de l'infection à VIH, une manifestation clinique du SK:*** avant la chimiothérapie (T0), la séroprévalence en IgG anti-HHV8 était de 93%, avec 60% de patients à risque de transmission. Douze mois plus tard, après une chimiothérapie ABV dont la moyenne des cures était de 4±2, on constatait que chez certains patients, la maladie continuait sa progression (51%). Cependant, cette séroprévalence tombait à 77%, où seulement 30% de patients restaient à risque de transmission.

***Séroprévalence du HHV8 dans la population d'étude en association avec les données épidémiologiques, biologiques et cliniques:*** en associant la séropositivité des anticorps anti-HHV8 et d'autres paramètres de l'étude, on avait les résultats suivants : voir table 1 et table 2.

**Tableau 1:** A T0, séroprévalence des anticorps IgG antiHHV8 chez les personnes présentant un SK et l'infection à VIH (SK^+^VIH^+^) et chez celles présentant seulement une infection à VIH (VIH^+^)

Variables	SK^+^VIH^+^				VIH^+^			
	n	IgG anti-HHV8(+)		*P* X^2^	n	IgG antiHHV8(+)		*P* X^2^
		n	%			n	%	
***sexe***								
Masculin	24	23	96	0,56 NS	10	9	90	0,25 NS
Féminin	19	17	89		4	2	50	
***Age (ans)***								0,07 NS
[21-30]	8	8	100	0,52 NS	1	0	0	
[31-40]	17	15	88		5	3	60	
[41-50]	9	8	89		4	4	100	
[51-60]	7	7	100		3	3	100	
[61-70]	2	2	100		1	1	100	
***Stade de la maladie SK***								-
St1&St2	34	32	94	0,69 NS	-	-	-	
St3&St4	9	8	89		-	-	-	
***Stade de l'infection à VIH***								0,078 NS
Stade précoce (T1&T2)	2	2	100	0,11 NS	3	1	34	
Stade avancé (T3&T4)	41	38	93		11	10	91	
***Taux de CD4*** ^***+***^ ***(cellules/mm*** ^***3***^ ***)***								0,081 NS
< 200	29	27	93	0,33 NS	5	2	40	
200 - 349	8	8	100		5	5	100	
350 - 499	4	3	75		4	4	100	
≥ 500	2	2	100		0	0	0	

IgG anti-HHV8(+) : positif aux anticorps X^2^ : khi deux, n : nombre de cas, % : pourcentage, p : p value, NS : non significatif

**Tableau 2:** Séroprévalence des anticorps IgG antiHHV8 chez les personnes présentant un SK et l'infection à VIH (SK^+^VIH^+^) et chez celles présentant seulement une infection à VIH (VIH^+^), à T12, douze mois plus tard

Variables	SK^+^VIH^+^				VIH^+^			
	n			P X^2^	n	IgG anti-HHV8(+)		P X^2^
		n	%			n	%	
**sexe**								0,27 NS
Masculin	24	19	79	0,46 NS	10	7	70	
Féminin	19	15	79		4	1	25	
**Age (ans)**								0,25 NS
[21-30]	8	7	88	0,007 NS	1	0	0	
[31-40]	17	13	76		5	2	40	
[41-50]	9	7	78		4	3	75	
[51-60]	7	6	86		3	2	67	
[61-70]	2	1	50		1	1	100	
**Stade de la maladie SK**								-
St1&St2	34	28	82	0,57NS	-	-	-	
St3&St4	9	6	67		-	-	-	
**Nombre de cures de la thérapie ABV**								-
[< 6]	25	18	72	0,57 NS	-	-	-	
[>6]	10	8	80		-	-	-	
[ 6]	8	8	100		-	-	-	
**Réponse au traitement de la maladie SK**								-
Rémission	2	2	100	0,56 NS	-	-	-	
Stable	10	8	80		-	-	-	
Partielle	9	6	67		-	-	-	
Progression	22	18	82		-	-	-	
**Stade de l'infection à VIH**								0,078 NS
Stade précoce (T1&T2)	2	2	100	0,7 NS	3	0	0	
Stade avancé (T3&T4)	41	32	78		11	8	73	
**Taux de CD4** ^**+**^ **(cellules/mm** ^**3**^ **)**								
< 200	11	7	64	0,36 NS	1	1	100	0,91 NS
200 - 349	15	14	94		6	3	50	
350 - 499	10	9	90		5	3	60	
≥ 500	7	4	57		2	1	50	

IgG anti-HHV8(+) : positif aux anticorps X^2^ : khi deux, n : nombre de cas, % : pourcentage, p : p value, NS : non significatif

Tableau 1 : on constatait que 51 personnes sur les 57 étaient toujours positives aux anticorps anti-HHV8, soit 90%. Bien que la tranche d'âge allant de 31 à 40 ans soit la plus représentée, c'est celle qui présentait le taux le plus bas de la séroprévalence en HHV8. 60% de personnes étaient immunodépressives sévères (ayant un taux de CD4^+^ <200). Une association significative de ces paramètres avec la séroprévalence en anticorps anti-HHV8 n'avait pas été observée.

Tableau 2 : on constatait que 42 personnes sur les 57 étaient toujours positives aux anticorps anti-HHV8, soit 74%. La tranche d'âge allant de 31 à 40 ans présentait toujours le taux le plus bas de la séroprévalence en HHV8. Seuls 21% de la population restaient immunodépressives sévères, avec aussi une baisse dans sa séroprévalence en anticorps anti-HHV8. Pour les personnes présentant une manifestation clinique du SK, 58% des malades avaient reçu moins de 6 cures, et l'on observait un fort taux de progression de cette maladie (51%), en réponse au traitement ABV. Une association significative de ces paramètres avec la séroprévalence en anticorps anti-HHV8 n'avait pas été observée. En comparant les deux tables, on constatait qu'au bout de 12 mois de suivi: la séroprévalence en anticorps anti-HHV8 baissait, quelque soit le groupe de patients; le nombre de personnes immunodépressives sévères baissait drastiquement; pas de manifestations cliniques de SK survenues chez les patients du groupe VIH^+^.

## Discussion

En début d'étude, la séroprévalence au HHV8 était de 90% et tombait à 74% douze mois plus tard. Ces taux restaient élevés, indépendamment des paramètres de l'étude, ce qui démontre une forte circulation de ce virus dans notre population d'étude, et rejoignait les résultats des études effectuées au Cameroun sur des personnes VIH^+^ et VIH^−^(séroprévalence à 70%) [14] et au Maroc sur des personnes présentant un SK non associé à une infection à VIH (séroprévalence à 92%) [15]. Cependant, ce taux est bien au-delà de celui observé dans d'autres études menées au Ghana (séroprévalence à 27%) [11] et au Nigéria (séroprévalence à 42%) [16] et encore au Cameroun sur les populations bantous et pygmées des régions du Sud et de l'Est camerounais (37,2%) [17]. Pour les patients VIH^+^ de notre cohorte, au moment de l'insertion à l'étude, la séroprévalence au HHV8 était de 79%, avec des valeurs de p non significatives pour les paramètres de l'étude. Il faudrait signaler que cette prévalence était tout de même moins élevée dans la tranche d'âge de 31 à 40 ans, en stade précoce de l'infection à VIH (stades 1 et 2), et chez les personnes ayant un taux bas de CD4^+^ (<200). Cela se compare avec la séroprévalence globale au HHV8 était de 65,6%, élevée dans les tranches d'âge allant de 26 à 45 ans et l'absence de différence significative entre les sexes au Ghana [11], ainsi que de la séroprévalence globale de 62 %, élevée chez les personnes avec CD4 <200, 4 fois plus élevée en HIV stade avancé au Nigéria [16]. Pour les patients SK^+^ VIH^+^, la séroprévalence au HHV8 était de 93%, valeur similaire à celle obtenue au Maroc (92%) chez des personnes présentant un SK non associé au VIH [15]. Douze mois plus tard, l'absence de manifestations cliniques du SK chez les patients VIH^+^ positifs à l'infection à HHV8 (et à des titres très élevés) et avec un taux de LTCD4^+^ <200 cellules/mm^3^. Cela est confirmé par la diminution de la séroprévalence du HHV8 dans la cohorte (de 74% à 57%). ainsi que le suggère Gallo en 1998 [16] qui suppose un rôle autre que l'immunodépression dans le développement du SK. Mais comme le démontre l'étude menée par Maglione et al, ces malades pourraient développer un SK lié au VIH/SIDA [16].

## Conclusion

La séroprévalence élevée en HHV8 dans la population d'étude, et dans celles menées au Cameroun dans notre revue [14, 17] suggère une endémicité de l'infection au Cameroun. La manifestation clinique de l'infection à HHV8 dans notre cohorte qu'est le sarcome de Kaposi paraît associée au stade avancé du VIH/SIDA, et un taux bas de CD4^+^. Un dépistage de l'infection par ce virus oncogène dans les populations à risque devrait être réalisé, surtout en présence d'immunodépression, ce qui peut permettre de prévenir le développement du SK lié au VIH/SIDA.
